# Quantitative determination and toxicity evaluation of 2,4-dichlorophenol using poly(eosin Y)/hydroxylated multi-walled carbon nanotubes modified electrode

**DOI:** 10.1038/srep38657

**Published:** 2016-12-12

**Authors:** Xiaolin Zhu, Kexin Zhang, Chengzhi Wang, Jiunian Guan, Xing Yuan, Baikun Li

**Affiliations:** 1 School of Environment, Northeast Normal University, Changchun 130117, P.R. China; 2 Department of Civil and Environmental Engineering, University of Connecticut, Storrs, CT 06269, USA

## Abstract

This study aimed at developing simple, sensitive and rapid electrochemical approach to quantitatively determine and assess the toxicity of 2,4-dichlorophenol (2,4-DCP), a priority pollutant and has potential risk to public health through a novel poly(eosin Y, EY)/hydroxylated multi-walled carbon nanotubes composite modified electrode (PEY/MWNTs-OH/GCE). The distinct feature of this easy-fabricated electrode was the synergistic coupling effect between EY and MWNTs-OH that enabled a high electrocatalytic activity to 2,4-DCP. Under optimum conditions, the oxidation peak current enhanced linearly with concentration increasing from 0.005 to 0.1 μM and 0.2 to 40.0 μM, and revealed the detection limit of 1.5 nM. Moreover, the PEY/MWNTs-OH/GCE exhibited excellent electrocatalytic activity toward intracellular electroactive species. Two sensitive electrochemical signals ascribed to guanine/xanthine and adenine/hypoxanthine in human hepatoma (HepG2) cells were detected simultaneously. The sensor was successfully applied to evaluate the toxicity of 2,4-DCP to HepG2 cells. The IC_50_ values based on the two electrochemical signals are 201.07 and 252.83 μM, respectively. This study established a sensitive platform for the comprehensive evaluation of 2,4-DCP and posed a great potential to simplify environmental toxicity monitoring.

2,4-dichlorophenol (2,4-DCP) is a typical chlorophenol compound widely used in herbicides, fungicides, and insecticides^1^. As a priority pollutant listed by the US Environment Protection Agency (EPA), 2,4-DCP seriously threatens environmental quality and public health^2^,^3^. Despite the maximum permissible concentration of 2,4-DCP in drinking water is set as 0.5 mg L^−1^, higher concentrations are usually found in polluted environments^4^,^5^. Until now, there has been no quantitative determination and toxicity evaluation method for early detection of 2,4-DCP. Due to its low allowable concentration and coexistence with interfering substances (e.g. phenols and metal ions), 2,4-DCP has been measured using UV spectrophotometry^6^, gas chromatography^7^, high-performance liquid chromatography^8^, and capillary electrophoresis^9^. However, most of these methods are complex, low sensitive, costly, and incapable of real-time monitoring. The electrochemical method newly developed is preferable owing to the simplicity, high sensitivity, low-cost, and continuous on-line detection^10^,^11^,^12^,^13^,^14^,^15^.

As for the *in vitro* toxicity evaluation of pollutants, some techniques such as the 3-(4,5-dimethylthiazol-2-yl)-2, 5-diphenyltetrazolium bromide (MTT) assay^16^, flow cytometry experiment^17^, and lactate dehydrogenase (LDH) release assay^18^ have been developed. Recently, cell-based electrochemical method that is simple, sensitive, rapid, label-free, and non-toxic has gained attention for toxicity assessment^19^,^20^,^21^. An *in-situ* electrochemical method was successfully developed and applied in drug screening and toxicity evaluation of heavy metals in our previous study^22^,^23^,^24^, which was based on the electrical signals of the intermediates of cellular purine metabolism, and objectively revealed the cell viability at the molecular level^25^. Graphene, carbon nanotubes, and threonine modified electrodes have been developed. Nevertheless, due to the insufficient sensitivity of these electrodes, only one electrochemical signal was detected at approximately +0.65 V corresponded to xanthine/guanine in human cancer cells. A carbon nanotubes and ionic liquid complex (MWNTs/IL) modified electrode captured two electrochemical signals ascribed to xanthine/guanine and hypoxanthine/adenine in MCF-7 cells at about +0.70 V and +1.0 V, respectively^26^. However, the electrode was fabricated by coating a MWNTs/IL paste on the electrode surface, which is difficult to control the thickness of the film. Therefore, it is critical to develop simple and stable electrodes for the sensitive determination of purine bases.

Carbon nanotubes (CNTs) have been used as the sensor material owing to their unique thermal, excellent optical and mechanical properties^27^. Although CNTs tend to entangle and hardly disperse in water because of their hydrophobic nature, they could be functionalized with different moieties to improve the solubility^28^,^29^. Hydroxylated multi-walled carbon nanotubes (MWNTs-OH) with π-π conjugating structure are a kind of functionalized CNTs that the structural defects can be obtained on the surface due to the sp^3^ hybridization of C-C bonds formed in the functionalization process^30^,^31^,^32^. These defects can not only improve the solubility but also significantly enhance the reactivity and electronic property. In the same time, Eosin Y (EY), as a xanthene dye containing bromine atoms, is widely applied as the biological stain, laser dye, fluorescent probe, and sensitizer due to its high stability, versatility, and light absorptivity^33^,^34^,^35^,^36^,^37^,^38^. Recently, EY was successfully applied for the fabrication of electrodes to monitor catechol and hydroquinone^39^, sodium dodecyl sulfate^40^, and hydroquinone and catechol^41^. EY acts as the excellent electron donor, and can generate stable redox active layers by the electrochemical polymerization. However, the development of EY as sensors is still at an early stage, and it has been rarely reported to interact with carbon nanomaterials. Given the compatible structure and physical properties of MWNTs-OH and EY, hybridization might be formed via π-π interaction, through which the EY/MWNTs-OH composite would presumably produce synergic effects and exhibit higher electrocatalytic activity by improving their unique potential.

In this study, a sensitive electrode with high electrocatalytic activity toward not only 2,4-DCP but also cellular purine bases was developed, so that simple, rapid, sensitive and quantitative determination and toxicity assessment would be realized to simplify the detection and evaluation process of 2,4-DCP (Fig. 1). The poly(eosin Y)/hydroxylated multi-walled carbon nanotubes hybrid material modified glass carbon electrode (PEY/MWNTs-OH/GCE) was developed. The electrode was characterized using emission scanning electron microscopy (SEM), electrochemical impedance spectroscopy (EIS), raman spectroscopy, and cyclic voltammetry (CV) technique. The electrochemical sensor was examined for the quantitative detection and toxicity assessment of 2,4-DCP on human hepatoma (HepG2) cells for the first time that have been proved as the sensitive and stable model organisms in environmental toxicology^42^.

## Results and Discussion

### Characterization of the PEY/MWNTs-OH/GCE

**Figure 2:**
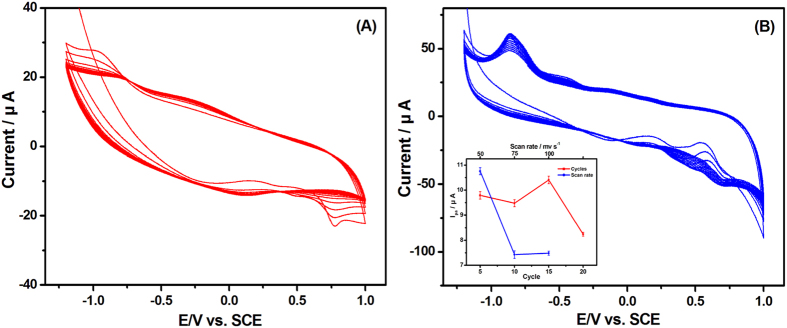
Electropolymerization curves of 500 μM EY in pH 5.0 PBS at (**A**) GCE and (**B**) MWNTs-OH/GCE. Inset: the influence of the cycle and scan rate on the oxidation peak current of 20 μM 2,4-DCP during the fabrication process of PEY/MWNTs-OH/GCE.

**Figure 3:**
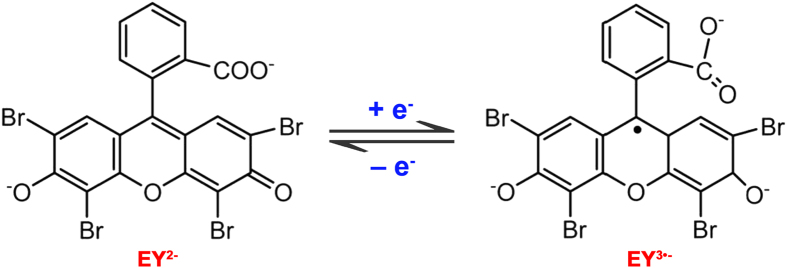
The structure and mechanism of electropolymerization process of EY.

The electrodeposition is a simple approach to immobilize organic molecules onto the electrode surface, which adjusts the surface properties including thickness, permeation, and charge transportation^43^. The successive CV curves for the electrochemical modification of PEY/GCE and PEY/MWNTs-OH/GCE were conducted over the potential range from −1.2 to +1.0 V. On the GCE (Fig. 2A), an anodic peak attributed to the oxidation of EY (EY^3·−^ − e^−^ → EY^2−^) appeared at +0.75 V and a cathodic peak attributed to the reduction (EY^2−^ + e^−^ → EY^3·−^) was observed at −0.87 V in the 1^st^ cycle. The peak current decreases with increase in number of cycles. On the MWNTs-OH/GCE (Fig. 2B), the background current was much larger than that of GCE, indicating the higher surface area of MWNTs-OH. Two oxidation and reduction peaks were found at the potential of +0.69 V, +0.45 V, −0.50 V, and −0.87 V, respectively. During the one-step electrodeposition process, the EY molecules acted as an excellent electron acceptor, which exist mainly in the form of EY^2−^ and could be reduced to EY^3−^ by the cleavage of the C=O bond on the benzene ring (Fig. 3). The obtained EY^3−^ could rapidly combine with the electrode surface^44^. The peak current was virtually constant after 15 cycles, indicating that the polymerization reached saturation at the MWNTs-OH/GCE. The thickness of the film had a significant contribution to the property of the PEY/MWNTs-OH/GCE. Thus, the influences of the cycle number and scan rate of EY on the electrocatalytic performance of PEY/MWNTs-OH/GCE were investigated (the inset of Fig. 2B). The oxidation peak current of 2,4-DCP reached to the maximum after 15 scans at the scan rate of 50 mV s^−1^. The peak current began to drop when the polymerizing cycles were more than 15. Thick films may prevent the electron transfer process and the oxidation process. Hence, the optimal electropolymerization cycles are selected as 15 cycles.

**Figure 4:**
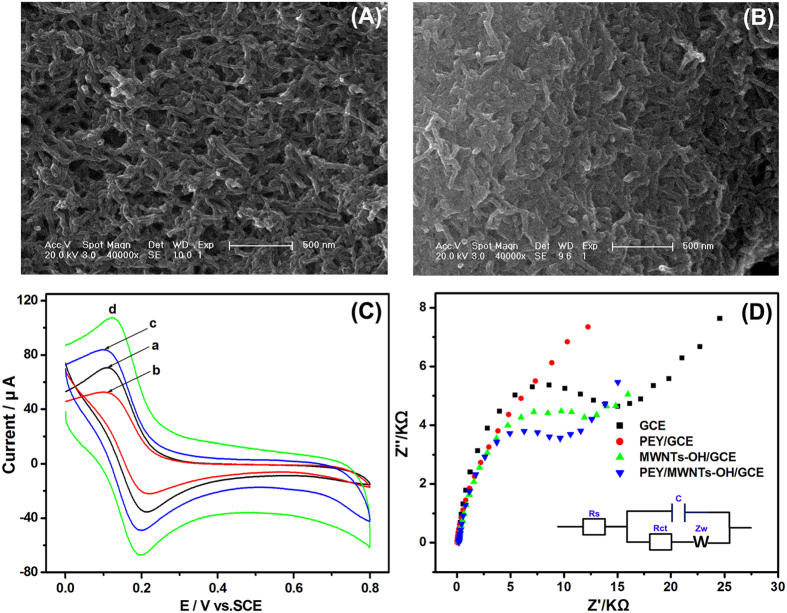
The SEM images of (**A**) MWNTs-OH and (**B**) PEY/MWNTs-OH; (**C**) CVs of GCE (a), PEY/GCE (b), MWNTs-OH/GCE (c), and PEY/MWNTs-OH/GCE (d) in 5.0 mM [Fe(CN)_6_]^3−/4−^ containing 0.1 M KCl; (**D**) EIS of different electrodes in 5.0 mM [Fe(CN)_6_]^3−/4−^ and 0.1 M KCl. Inset: the equivalent circuit model to fit the impedance data.

The surface morphologies of each layer were characterized by SEM. Randomly oriented MWNTs-OH was detected with interconnected tubular structures (Fig. 4A), which was the characteristic of carbon nanotubes^45^. After the introduction of EY molecules, the EY polymer was uniformly distributed over the MWNTs-OH (Fig. 4B). The homogeneous and uniform film with three-dimensional network structure was produced, demonstrating that the EY could be modified effectively on the surface with the electrodeposition method. The highly conjugated PEY/MWNTs-OH composite exhibited high surface area, and thus providing more sites for the accumulation of the target molecule.

Then the CVs of different electrodes were studied in 5.0 mM [Fe(CN)_6_]^3−/4−^ containing 0.1 M KCl (Fig. 4C). The electrochemically active surface area can be obtained according to the following equation^46^:


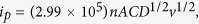


where *i*_*p*_ is the peak current, *n* is the number of electrons involved in the redox reaction of Fe(CN)_6_^3−/4−^ (*n* = 1), *A* is the electroactive surface area, *C* is the reactant concentration, *D* is the diffusion coefficient (6.30 × 10^6^ cm^2^ s^−1^), and *v* is the scan rate. The active surface areas for MWNTs-OH/GCE and PEY/MWNTs-OH/GCE were calculated as 0.079 cm^2^ and 0.108 cm^2^, respectively, which further confirmed that the PEY film increased the surface area of the MWNTs-OH/GCE.

EIS can provide details on the interfacial properties of the electrode interface during the fabrication process. The semicircular portion at higher frequencies represents the electron transfer-limited process, and the diameter corresponds to the electron transfer resistance (R_ct_)^47^. Figure 4D shows the typical Nyquist plots recorded at frequencies ranging from 0.01 to 10^5^ Hz in 0.1 M KCl containing 5.0 mM [Fe(CN)_6_]^3−^/^4−^ as the electrochemical redox probe. The inset shows the equivalent circuit model to fit the impedance data. R_s_, C, and W represented the solution resistance, pure capacitance, and Warburg impedance, respectively. The EIS at the bare GCE displayed a well-defined semicircle, with a huge interfacial R_ct_ of 14.3 KΩ. The increase in the R_ct_ of PEY/GCE suggested a successful modification of EY on the GCE surface. The R_ct_ decreased dramatically to 12.1 KΩ after the introduction of MWNTs-OH on the GCE, confirming an excellent electron conducting ability of MWNTs-OH. The lower R_ct_ of PEY/MWNTs-OH/GCE (9.2 KΩ) demonstrated the enhanced electron transfer reaction, which was mainly attributed to the synergistic effect between EY and MWNTs-OH.

**Figure 5:**
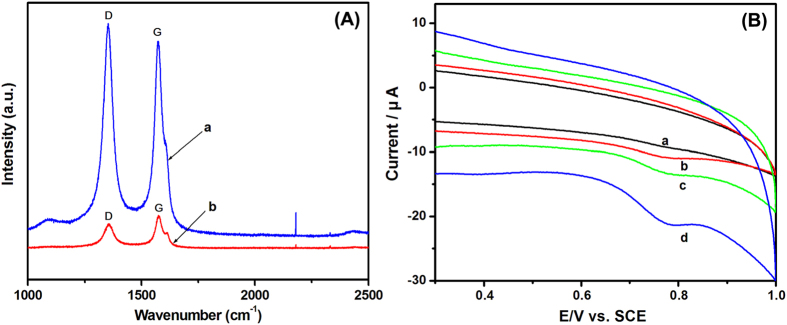
(**A**) Raman spectrum of MWNTs-OH (a) and PEY/MWNTs-OH nanocomposite (b); (**B**) CVs of GCE (a), PEY/GCE (b), MWNTs-OH/GCE (c), and PEY/MWNTs-OH/GCE (d) in 0.1 M pH 3.0 PBS containing 20 μM 2,4-DCP.

Raman spectroscopy is a useful tool to obtain structural information of carbonaceous materials^48^. The D band corresponds to the disordered structural defects and the G band is due to the first-order scattering of the E_2g_ mode for sp^2^ carbon lattice. The relative intensity ratio of D band to G band (*I*_D_/*I*_G_) can be used to examine the disorder and defects. The decrease of *I*_D_/*I*_G_ indicated the increase in the average size of sp^2^ domains^49^. PEY/MWNTs-OH (Fig. 5A, curve b) exhibits the D band at 1357 cm^−1^ and the G band at 1580 cm^−1^ with intensities lower than that of MWNTs-OH (Fig. 5A, curve a). The *I*_D_/*I*_G_ value of MWNTs-OH was estimated to be 1.09. In the case of PEY/MWNTs-OH, the *I*_D_/*I*_G_ value decreased to 0.78, which implied the successful formation of the PEY/MWNTs-OH hybrids and an efficient π-π interaction between them.

### Voltammetric behavior of 2,4-DCP at the PEY/MWNTs-OH/GCE

The CVs of 2,4-DCP at different electrodes were investigated (Fig. 5B). No peak was observed at the GCE (curve a), which was consistent with the previous study^13^. A broad oxidation peak appeared at the PEY/GCE (curve b) but it was too weak to distinguish. In the case of MWNTs-OH/GCE, a peak at +0.78 V was observed (curve c), while a well-defined anodic peak was obtained at +0.77 on PEY/MWNTs-OH/GCE (curve d). There was no corresponding reduction peak in the inverse scan, which was the characteristic of an irreversible electrode process. The high background current of PEY/MWNTs-OH/GCE reflected its effective surface area. In addition, the peak current was 5.2 times that of PEY/GCE and 2.9 times that of MWNTs-OH/GCE. Considering that graphene (Gr) was also an ideal material which can be coupled with organic dye molecules through the electron cloud overlap, the PEY/MWNTs-OH/GCE was compared with PEY/Gr/GCE (Fig. S1). The oxidation peak current of 2,4-DCP at PEY/MWNTs-OH/GCE was 2.1 times as large as PEY/Gr/GCE. The above results were caused by two reasons. First, MWNTs-OH increased the electronic conductivity, and the well-distributed poly(eosin Y) film improved the surface area for high adsorptive capability for 2,4-DCP. Second, the synergistic effect between PEY and MWNTs-OH increased the electrocatalytic activity as well as promoted the electron-transfer rate.

**Figure 6:**
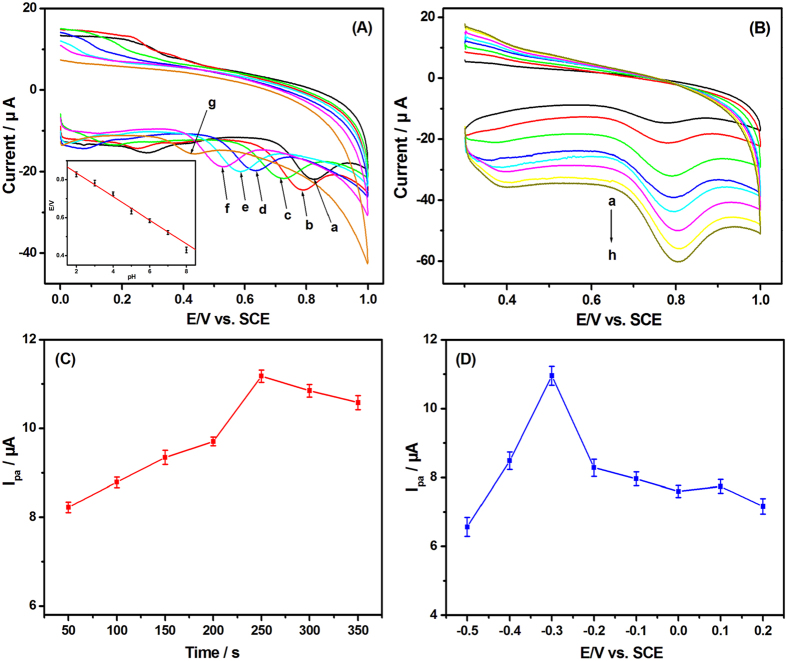
Influence of (**A**) pH (a–g: 2, 3, 4, 5, 6, 7, and 8), (**B**) scan rate (a–h: 30, 50, 80, 100, 120, 150, 180, and 200 mV s^−1^), (**C**) accumulation time, and (**D**) accumulation potential on the oxidation peak current of 20 μM 2,4-DCP at the PEY/MWNT-OH/GCE. Inset: relationships between pH and the peak potential.

The experimental parameters for the detection of 2,4-DCP using the PEY/MWNTs-OH/GCE were optimizated. The effects of pH on the oxidation peak current (*I*_*p*_) and peak potential (*E*_p_) of 2,4-DCP at the PEY/MWNTs-OH/GCE were studied at the pH range of 2.0–8.0 (Fig. 6A). The *I*_*p*_ reached to the maximum at pH 3.0 and declined obviously at pH 4.0. There was no significant change in peak current from pH 4.0 to 7.0, but the *I*_*p*_ decreased sharply at the pH value of 8.0, which was related to the electrochemical mechanism of 2,4-DCP. The lower pH promoted the ionization process of 2,4-DCP at the initial stages of the electrochemical oxidation, in which some phenol hydroxyl radicals were generated^50^. Meanwhile, the *E*_p_ of 2,4-DCP shifted linearly toward negative potential values with increasing the pH between 2.0 and 8.0 (inset of Fig. 6A). The linear regression equation was *E*_*p*_ = −0.0662 pH + 0.975 (R = 0.993). The slope (−0.0662 V pH^−1^) revealed that equal numbers of electron and proton are involved in the reaction process of the 2,4-DCP^51^. Therefore, the optimum pH was selected as 3.0.

The effect of scan rate on the electrochemical characteristics of 2,4-DCP was then studied. The *I*_p_ of 2,4-DCP increased with the scan rate in the range from 30 to 200 mV s^−1^ (Fig. 6B). The linear relationship between *I*_p_ and the square root of the scan rate (*v*^1/2^) can be expressed as *I*_p_ (μA) = 2.227 ν^1/2^ (mVs^−1^) − 6.692 (R = 0.997), indicating that the electron transfer process was controlled by diffusion. Meantime, the *E*_p_ shifted positively with the increasing in the scan rate. The *E*_p_ changed linearly versus the natural logarithm of scan rate (ln *v*) with a linear regression equation of *E*_p_ (V) = 0.0188 ln *ν* (mV s^−1^) + 0.703 (R = 0.996). According to Laviron’s theory^52^, the relationship between *E*_p_ and ln *v* in the irreversible electrode process could be described as


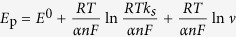


where *v* is the scan rate, *α* is the electron transfer coefficient, *k*_*s*_ is standard rate constant, and *R*, *T*, and *F* have their usual meanings. *α* is presumed as 0.4 in the irreversible electrode process^53^, and *αn* was easily calculated from the plot of *E*_*p*_ versus ln *v*. The electron transfer number *n* is calculated to be 2. Thus, the oxidation process of 2,4-DCP on PEY/MWNTs-OH/GCE is a two-electron and two-proton process^5^.

Moreover, the peak current is related to the amount of phenols accumulated on the modified electrode^54^. The influence of accumulation time and potential was investigated. The peak current of 2,4-DCP increased noticeably with raised accumulation time within 250 s (Fig. 6C). When the accumulation time was longer than 250 s, the peak current dropped slightly, indicating the 2,4-DCP reached the level of saturation. Additionally, the influence of accumulation potential was investigated (Fig. 6D), in which the maximum current was obtained at −0.30 V. Thus the optimal accumulation conditions were chosen as 250 s and −0.30 V for the electrochemical detection.

### Quantitative determination of 2,4,-DCP using the PEY/MWNTs-OH/GCE

**Figure 7:**
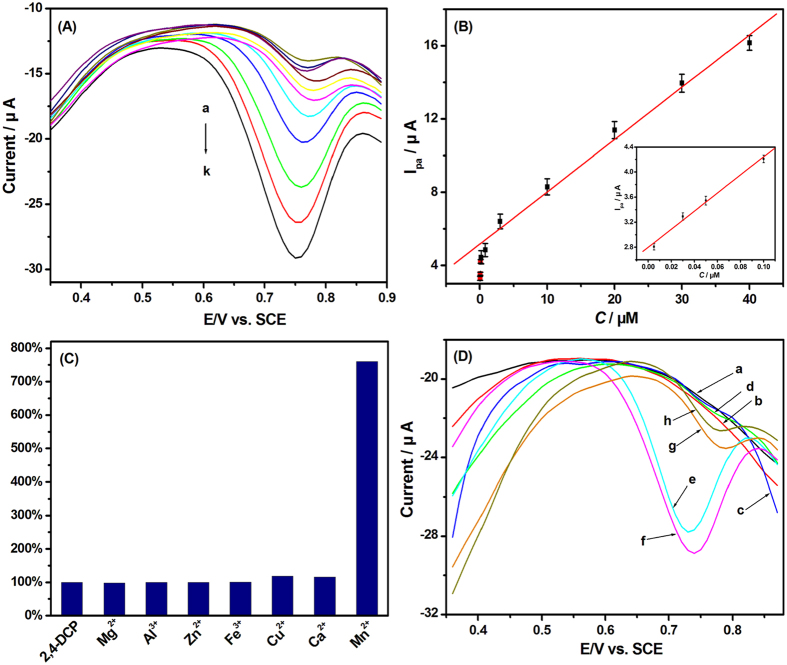
(**A**) DPVs of PEY/MWNTs-OH/GCE in pH 3.0 PBS with different 2,4-DCP concentrations (a–k: 0.005, 0.03, 0.05, 0.1, 0.2, 0.8, 3, 10, 20, 30, and 40 μM); (**B**) The relationship between peak current and the concentration of 2,4-DCP; (**C**) Column chart of the peak current of 20 μM 2,4-DCP containing 1 mM metal ions; (**D**) DPVs of 20 μM phenols.

Under the optimum conditions, the oxidation peak currents of 2,4-DCP at different concentrations on the PEY/MWNTs-OH/GCE were investigated by DPVs (Fig. 7A). The peak current of 2,4-DCP increased linearly at the concentration ranges of 5.0 × 10^−9^–1.0 × 10^−7^ M and 2.0 × 10^−7^–4.0 × 10^−5^ M. The linear regression equations were *I*_*p*_ (μA) = 14.431 c (μM) + 2.795 and *I*_*p*_ (μA) = 0.291 c (μM) + 5.026 with coefficients of 0.993 and 0.994, respectively (Fig. 7B). The lowest detection limit was calculated as 1.5 × 10^−9^ M (S/N = 3). Compared with other electrodes reported^5^,^10^,^11^,^12^,^13^,^14^,^15^, the PEY/MWNTs-OH/GCE electrode exhibited relatively wide linear range and low detection limit (Table 1), and thus was a promising sensor for the sensitive determination of 2,4-DCP.

The selectivity of the PEY/MWNTs-OH/GCE was investigated by adding several metal ionsand structurally similar phenols. 50-fold Mg^2+^, Al^3+^, Zn^2+^, Fe^3+^, Cu^2+^, and Ca^2+^ had negligible effects on the electrochemical oxidation of 2,4-DCP at PEY/MWNTs-OH/GCE except for Mn^2+^ (Fig. 7C). As shown in Fig. 7D, no peak was observed for hydroquinone (a) and catechol (b) between +0.3 and +0.9 V. Although PEY/MWNTs-OH/GCE showed the electrocatalytic activity to o-nitrophenol (c), p-nitrophenol (d), 2,4,6-trichlorophenol (g) and pentachlorophenol (h), the potential differences between these phenols and 2,4-DCP were lager than 60 mV. Thus they had no influence on the peak current of 2,4-DCP (f). However, a remarkable peak was observed for 2-chlorophenol (e), and its peak potential was similar to that of 2,4-DCP. Therefore, the PEY/MWNTs-OH/GCE is applicable for the detection of 2,4-DCP in the absence of Mn^2+^ and 2-CP.

The reproducibility was investigated by six measurements of 20.0 μM 2,4-DCP with the same PEY/MWNTs-OH/GCE. The relative standard deviation (RSD) was 3.74%. Additionally, six electrodes were examined at 20.0 μM 2,4-DCP and the RSD was 4.77%. These results proved that the sensor possessed excellent reproducibility and repeatability.

Furthermore, the analytical reliability and application potential of PEY/MWNTs-OH/GCE was investigated to determine 2,4-DCP in real water samples obtained from Yitong River (Changchun, China). The recoveries ranged from 93.2% to 105.6% (Table 2) indicating the excellent reliability and applicability of PEY/MWNTs-OH/GCE.

### Electrochemical behavior of HepG2 cells at the PEY/MWNTs-OH/GCE

**Figure 8:**
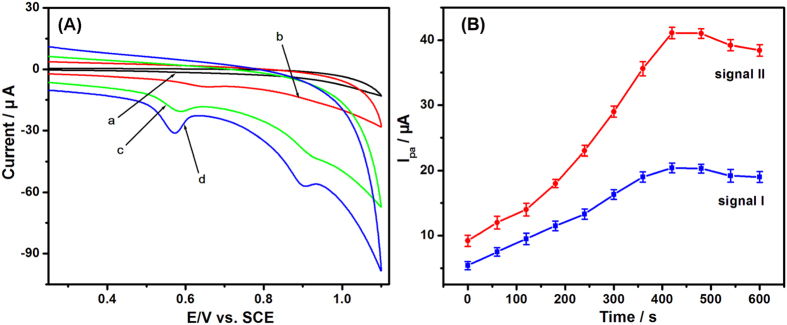
(**A**) CVs of HepG2 cell suspension at the (a) GCE, (b) PEY/GCE, (c) PEY/MWNTs-OH/GCE, and (d) PEY/MWNTs-OH/GCE. **(B)** Influence of accumulation time on the electrochemical signals of HepG2 cell suspension. Cell inoculation concentration: 4.0 × 10^5^ cells mL^−1^.

The potential application of the PEY/MWNTs-OH/GCE in HepG2 cell suspension (3.0 × 10^6^ cells mL^−1^) was explored (Fig. 8A). No peak appeared on the bare GCE (curve a). A broad oxidation peak was observed at about +0.65 V at the PEY/GCE (curve b). For MWNTs-OH/GCE (curve c), an oxidation peak was obtained at +0.60 V, and an oxidation peak appeared at +0.92 V, whereas, it was too weak to recognize. With the PEY/MWNTs-OH/GCE in HepG2 cell suspension (curve d), the background current was greater than other electrodes, suggesting the more effective surface area. Meanwhile, two well-defined oxidation peaks at +0.59 V and +0.90 V attributed to xanthine/guanine, and hypoxanthine/adenine^22^,^23^,^26^ were observed. The oxidation potentials shifted to less positive ones and the maximum peak currents were obtained. These results implied that the PEY/MWNTs-OH film possessed unique electrocatalytic activities toward the purine bases in HepG2 cells, and could promote electron transfer reactions. The influence of accumulation condition on the electrochemical signal was investigated (Fig. 8B), and 420 s was chosen as the best accumulation time.

**Figure 9:**
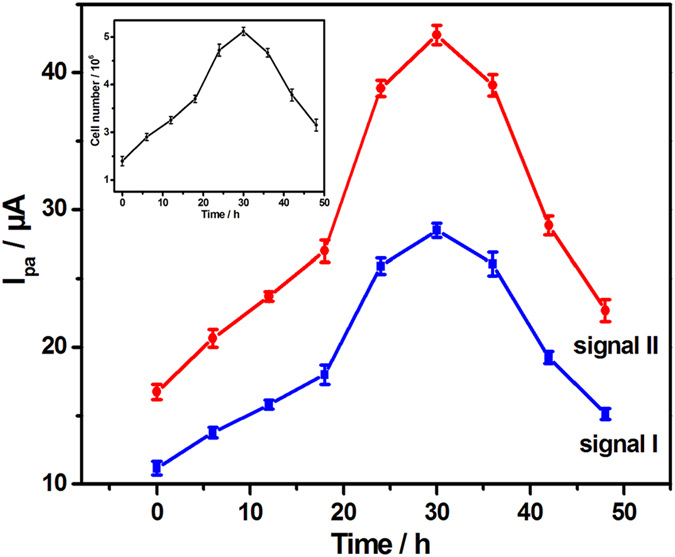
Growth curves of the HepG2 cells depicted by the PEY/MWNTs-OH/GCE and the cell counting method (inset).

The two electrochemical signals on PEY/MWNTs-OH/GCE were applied to describe the growth of HepG2 cells (Fig. 9). The peak currents increased gradually with the culture time within 30 h owing to the proliferation of cells. Then the peak currents reduced substantially because of the cell death caused by the lack of nutrients. These results well corresponded with the cell counting method (inset of Fig. 9), implying that the PEY/MWNTs-OH/GCE can be applied to monitor cell growth in real-time mode.

### Cytotoxicity assessment of 2,4-DCP on HepG2 cells

**Figure 10:**
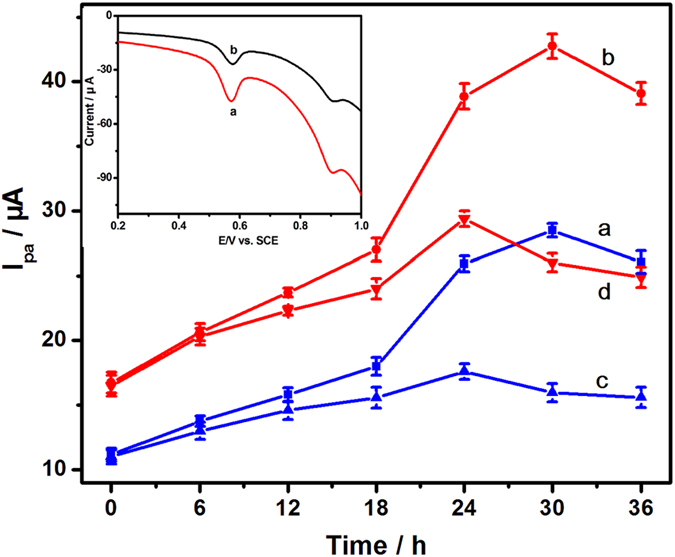
Dependence of the peak current of HepG2 cells on the culture time in the absence of 2,4-DCP based on electrochemical signal I (a) and signal II (b), and in the presence of 2,4-DCP based on electrochemical signal I (c) and signal II (d). Inset: CVs of HepG2 cells cultured for 30 h in the (a) absence and (b) presence of 2,4-DCP.

The electrochemical behaviors of HepG2 cells treated by 2,4-DCP at different times were investigated using PEY/MWNTs-OH/GCE (Fig. 10). Compared with control groups (curves a and b), the electrochemical signal I (curve c) and signal II (curve d) of 2,4-DCP treated groups increased slowly until 24 h and then decreased ascribed to the toxicity of 2,4-DCP. Meanwhile, the peak currents of 2,4-DCP were lower than those of the control group, indicating the inhibitory effects of 2,4-DCP on HepG2 cells. A notable decrease in the electrochemical response was observed after treated by 2,4-DCP for 30 h, and the cytotoxicity reached to the maximum (inset of Fig. 10). Therefore, 30 h was chosen as the best 2,4-DCP-treated duration.

**Figure 11:**
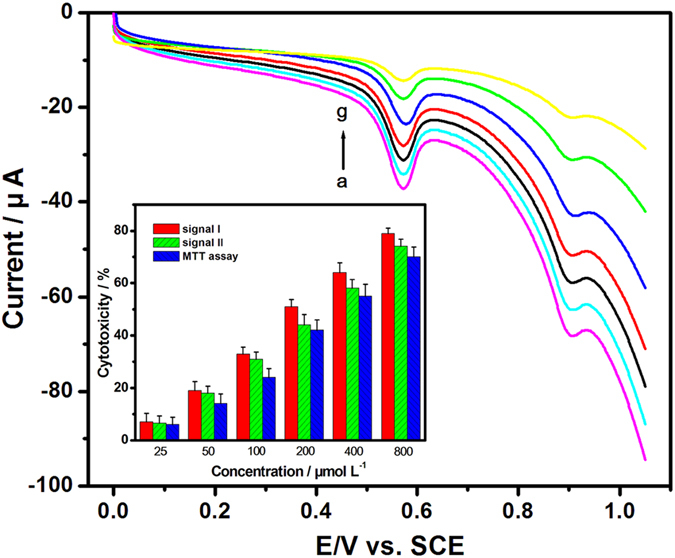
CVs obtained by PEY/MWNTs-OH/GCE with HepG2 cells exposed to 2,4-DCP with different concentrations (a-g: 0, 25, 50, 100, 200, 400, 800 μM). Inset: cytotoxicity of 2,4-DCP obtained by the PEY/MWNTs-OH/GCE and the MTT assay.

The electrochemical behaviors of HepG2 cells after treated by 2,4,-DCP with different concentrations for 30 h were studied using PEY/MWNTs-OH/GCE (Fig. 11). 2,4,-DCP exhibited significant cytotoxicity with a concentration-dependent pattern (inset of Fig. 11). The cytotoxicity (*Y*) was linear to the logarithm of 2,4,-DCP concentration (*X*) with the linear regression equations of *Y* = 46.35 *X* − 56.76 (R = 0.990) for signal I and *Y* = 42.50 *X* − 52.12 (R = 0.993) for signal II. The IC_50_ values based on the two electrochemical signals were 201.07 and 252.83 μM, respectively, reflecting that 2,4,-DCP had greater impacts on xanthine/guanine than those on hypoxanthine/adenine. It also confirmed the toxicity differences of 2,4-DCP to purine metabolism, which had a significance for the study of the cellular physiological process. 2,4,-DCP has been proved as a potential environmental endocrine disruptor and oxidative damage inducer^55^, and can change the antioxidant enzyme activities and induce oxidative stress, a mechanism responsible for DNA damage and inhibition of cell growth^56^,^57^. The lower level of cell viability reduced the signals on PEY/MWNTs-OH/GCE, which related to the cellar physiological changes.

Furthermore, the conventional MTT assay was applied to compare with the result of the PEY/MWNTs-OH/GCE. The linear regression equation obtained by the MTT assay was *Y* = 41.51 *X* − 53.44 (R = 0.993) and the IC_50_ value was 309.03 μM. The result verified the sensitivity of the electrochemical method on the toxicity evaluation of 2,4-DCP.

## Conclusion

A novel electrochemical sensing platform was constructed in a simple and green way. The PEY/MWNTs-OH/GCE significantly facilitated the electron transfer efficiency and possessed excellent electrocatalytic activity. Under the optimal condition, it exhibited a high sensitivity, wide linear range, excellent reproducibility and good stability toward 2,4-DCP. The easy-fabricated electrode developed was also employed in real water sample analysis and showed high accuracy. Moreover, the electrochemical behavior of HepG2 cells was investigated by the PEY/MWNTs-OH/GCE. The cytotoxicity of 2,4-DCP was successfully evaluated by this electrode. This study constituted a promising tool for 2,4-DCP analysis and toxicity evaluation, and revealed a simple sensitive electrochemical approach in the field of environmental monitoring and toxicology.

## Experimental Section

### Materials and chemicals

The MWNTs-OH (Nanjing XFNANO Materials TECH Co., Ltd.) was 20–40 nm in diameter with purity higher than 97%. The minimum essential medium (MEM), defined fetal bovine serum (FBS) (Gibco Co., USA), penicillin, streptomycin, trypsin (Sigma, Co., USA) were used for cell culture. Dimethyl formamide and eosin Y (EY) (J&K Chemical Ltd., China) were applied for electrode fabrication. 2,4-DCP (J&K Chemical Ltd., China) was used as the target compound. Phosphate-buffered solutions (PBS) were prepared by mixing stock solutions of 0.1 M Na_2_HPO_4_ and 0.1 M KH_2_PO_4_.

### Apparatus

The electrochemical measurements including cyclic voltammetry (CV) and differential pulse voltammetry (DPV) were performed on the CHI 760E electrochemical workstation (Shanghai CH Instruments, China). A three-electrode system was used, which consisted of a modified GCE as the working electrode, a saturated calomel electrode (SCE) as the reference electrode, and a platinum wire as the auxiliary electrode. The surface morphology of the nanocomposite was observed using a JEOL 6340F Scanning electron microscope (SEM). Electrochemical impedance spectroscopy (EIS) was performed using the PARSTAT 2273 potentiostat (Princeton applied research, USA). The Raman scattering spectra was obtained by the Jobin-Yvon HR 800 instrument with an Ar^+^ laser source of 488 nm wavelength. The pH of the solution was measured with a PB-10 pH-meter (Sartorius Co., Germany).

### Preparation of the PEY/MWNTs-OH/GCE

The glass carbon electrode (GCE) was sequentially polished using 1.0, 0.3 and 0.05 μm alumina slurry and rinsed successively with double-distilled water and ethanol solution. After 10.0 mg MWNTs-OH was dispersed with 10.0 mL dimethyl formamide using ultrasonic agitation, 5.0 μL MWNTs-OH dispersion was dropped onto GCE and dried under the infrared lamp to obtain the MWNTs-OH/GCE. Then the electropolymerization of EY was performed in PBS (pH 5.0) containing 500 μM EY and 0.3 mM KNO_3_ by applying the potential (−1.2 V to +1.0 V) at 50 mV s^−1^ for 15 cycles (Fig. S2). The obtained electrode was termed as PEY/MWNTs-OH/GCE.

### Cells culture and treatment

The HepG2 cells (COBIOER Biosciences Co., Ltd.) were cultured in 60 mm cell culture dish in the minimum essential medium (MEM) that was supplemented with 10% FBS, penicillin (100 μg mL^−1^), streptomycin (100 μg mL^−1^), 1% non-essential amino acids, and 1% sodium pyruvate at 37 °C in a humidified 5% CO_2_. For the toxicity investigation, the growth medium was replaced with the medium containing 2,4-DCP. The *in-situ* cell collection was conducted as our previous study^23^. Briefly, the PBS was added to cells after the medium was removed, and then the mixture was heated in the water bath at 50 °C for 30 min to obtain the HepG2 cell suspension.

### Electrochemical determination

For the electrochemical detection of 2,4-DCP, the CV was employed between +0.1 and +1.0 V with the scan rate of 50 mV s^−1^. The DPV was performed with the following parameters: increment potential, 4 mV; pulse amplitude, 0.05 V; pulse width, 0.05 V; pulse period, 0.2 s; sample width, 50 ms. The *in vitro* toxicity of 2,4-DCP to HepG2 cells was investigated from 0.0 to +1.1 V with the scan rate of 50 mV s^−1^. After each measurement, the PEY/MWNTs-OH/GCE was scanned for five cycles between 0.0 and +1.1 V in PBS and rinsed thoroughly with double-distilled water.

### The MTT assay

HepG2 cells (1.2 × 10^4^ cells mL^−1^) in medium alone (200 μL) or the medium containing 2,4-DCP (200 μL) were added to the 96-well microtitre plates. 20 μL 5 mg mL^−1^ MTT was added to each well after the incubation at 37 °C for 30 h. The medium containing MTT was removed after 4 hours, and 150 μL sodium dodecyl sulfate was added. The measurement was registered on an ELX800 Microplate Reader (BioTek Instruments, Inc., USA) and determined by the absorbance values at 490 nm.

## Additional Information

**How to cite this article**: Zhu, X. *et al*. Quantitative determination and toxicity evaluation of 2,4-dichlorophenol using poly(eosin Y)/hydroxylated multi-walled carbon nanotubes modified electrode. *Sci. Rep.* **6**, 38657; doi: 10.1038/srep38657 (2016).

**Publisher's note:** Springer Nature remains neutral with regard to jurisdictional claims in published maps and institutional affiliations.

## Supplementary Material

Supplementary Information

## Figures and Tables

**Figure 1:**
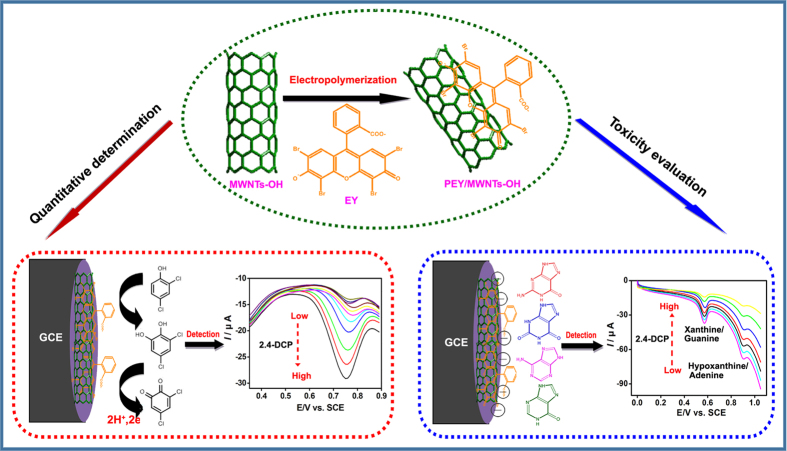
Schematic illustration of PEY/MWNTs-OH/GCE and the mechanism for detecting 2,4-DCP.

**Table 1:** Comparison of different electrodes for detection of 2,4-DCP.

Electrode	Technique	Linear range(μM)	Detection Limit (μM)	Ref.
HRP/MWNTs/GCE	Amperometry	1.0–100	0.38	10
Graphene/HRP/GCE	Amperometry	0.01–13.0	0.005	11
Nafion/MWNT/GCE	Amperometry	0.1–100	0.037	12
MB-AG/GCE	Amperometry	12.5–208	2.06	13
Nafion/PSS-GN-CTAB/GCE	LSV	0.01–2	0.002	14
MIP/GCE	DPV	5–100	1.6	15
CS/CDs-CTAB/GCE	DPV	0.04–8	0.01	5
PEY/MWNT-OH/GCE	DPV	0.005–0.1 0.2–40	0.0015	This work

HRP, horseradish peroxidase; MB-AG, myoglobin and agarose; PSS-GN-CTAB, poly(sodium-styrenesulfonate) functionalized graphene/cetyltrimethylammonium romide; MIP, molecularly imprinted polymers; CS/CDs-CTAB, chitosan/carbon dots and hexadecyltrimethyl ammonium bromide.

**Table 2:** Recovery results for 2,4-DCP in real water samples.

Sample	Added (μM)	Found (μM)	Recovery (%, n = 3)
Upstream watersample 1	0.1	0.099	99.0
Upstream watersample 2	1	0.932	93.2
Upstream watersample 3	5	5.28	105.6
Upstream watersample 4	20	20.4	102.0
Downstream watersample 1	0.1	0.095	95.0
Downstream watersample 2	1	0.988	98.8
Downstream watersample 3	5	4.88	97.6
Downstream watersample 4	20	20.5	102.5
