# Author Correction: Quantitative determination and toxicity evaluation of 2,4-dichlorophenol using poly(eosin Y)/hydroxylated multi-walled carbon nanotubes modified electrode

**DOI:** 10.1038/s41598-021-84930-2

**Published:** 2021-03-09

**Authors:** Xiaolin Zhu, Kexin Zhang, Chengzhi Wang, Jiunian Guan, Xing Yuan, Baikun Li

**Affiliations:** 1 School of Environment, Northeast Normal University, Changchun, 130117 P.R. China; 2 Department of Civil and Environmental Engineering, University of Connecticut, Storrs, CT 06269 USA

Correction to: *Scientific Reports* https://doi.org/10.1038/srep38657, published online 12 December 2016

In this Article, Baikun Li is incorrectly listed as corresponding author. The correct corresponding author for this Article is Xing Yuan.

Correspondence and request for materials should be addressed to yuanx@nenu.edu.cn.

