# Supplementary material for: Quantitative determination and toxicity evaluation of 2,4-dichlorophenol using poly(eosin Y)/hydroxylated multi-walled carbon nanotubes modified electrode
Source: Sci Rep. 2016 Dec 12;6:38657. doi: 10.1038/srep38657 (PMC5150580; doi:10.1038/srep38657)
Supplement: Supplementary Information [file srep38657-s1.doc]

**Supporting information**

### Quantitative determination and toxicity evaluation of 2,4-dichlorophenol using poly(eosin Y)/hydroxylated multi-walled carbon nanotubes modified electrode

Xiaolin Zhu^1^, Kexin Zhang^1^, Chengzhi Wang^1^, Jiunian Guan^1^, Xing Yuan^1^,* & Baikun Li^2^,*

^1^School of Environment, Northeast Normal University, Changchun 130117, P.R. China.

^2^Department of Civil and Environmental Engineering, University of Connecticut, 261 Glenbrook Road, Unit 2037, CT 06269, USA.

*These authors contributed equally to this work. Correspondence and requests for materials should be addressed to X.Y. (email: yuanx@nenu.edu.cn) or B.-k.L. (email: baikun@engr.uconn.edu)


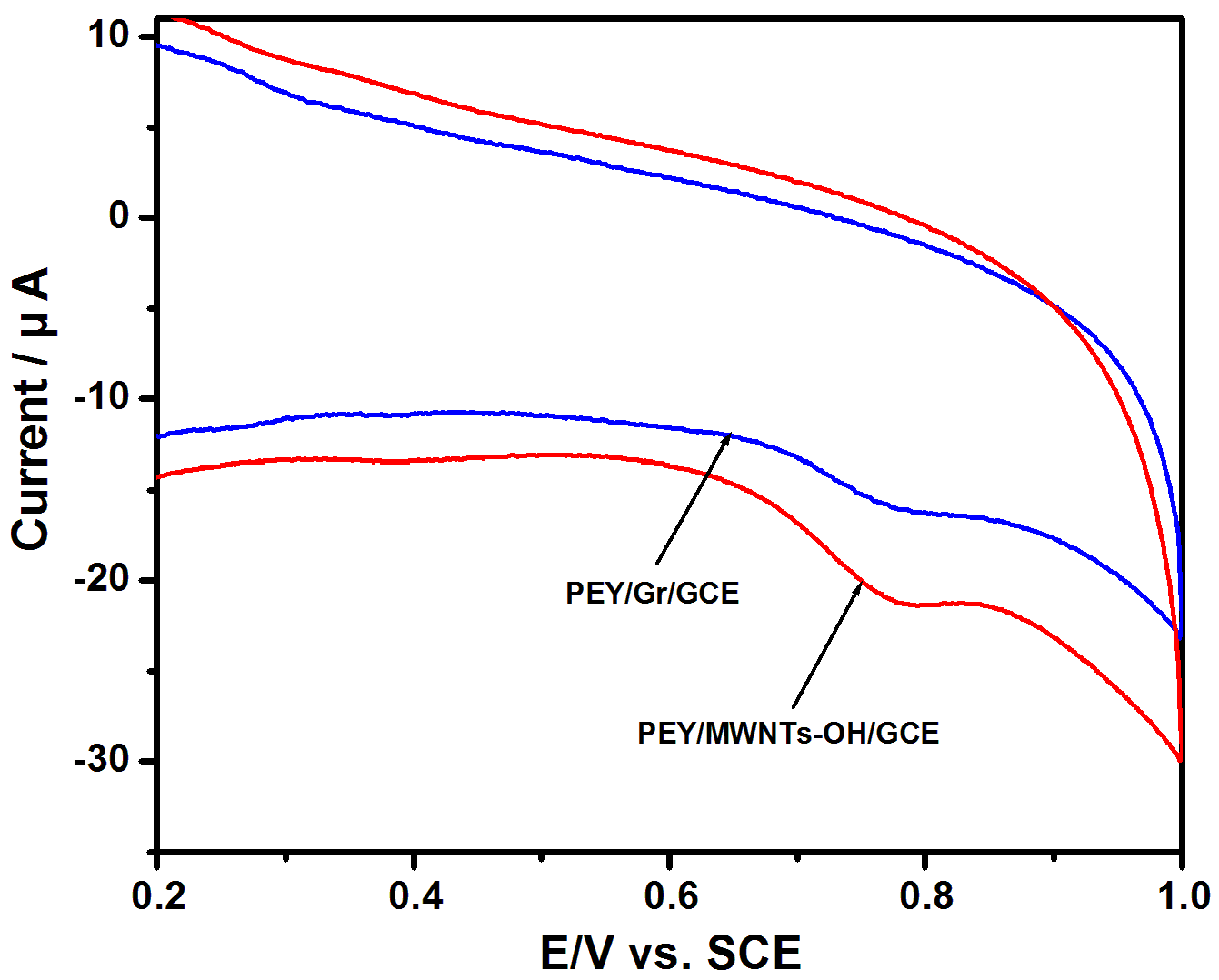


**Fig. S1.** CVs of PEY/MWNTs-OH/GCE and PEY/Gr/GCE in 0.1 M pH 3.0 PBS containing 20 μM 2,4-DCP.


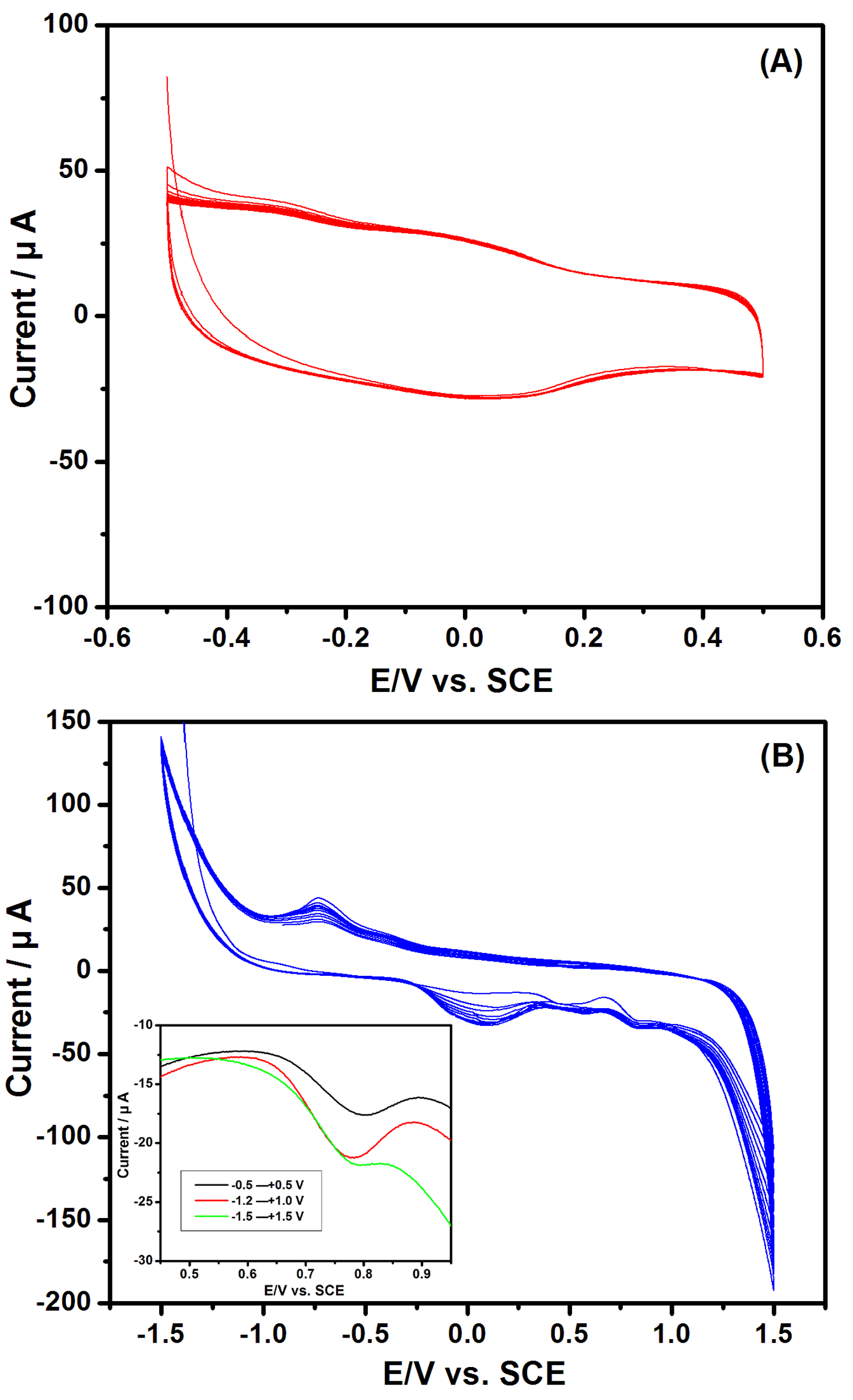


**Fig. S2.** Electropolymerization curves of 500 μM EY in pH 5.0 PBS at MWNTs-OH/GCE within the potential window of -0.5—+0.5 V (A) and -1.5—+1.5 V (B). Inset: DPVs of PEY/MWNTs-OH/GCEs obtained by different potential windows in 0.1 M pH 3.0 PBS containing 20 μM 2,4-DCP.
